# Structural Health Monitoring of Above-Ground Storage Tank Floors by Ultrasonic Guided Wave Excitation on the Tank Wall

**DOI:** 10.3390/s17112542

**Published:** 2017-11-04

**Authors:** Premesh S. Lowe, Wenbo Duan, Jamil Kanfoud, Tat-Hean Gan

**Affiliations:** 1 Brunel Innovation Centre (BIC), Granta Park, Great Abington, Cambridgeshire CB21 6AL, UK; wenbo.duan@brunel.ac.uk (W.D.); jamil.kanfoud@brunel.ac.uk (J.K.); 2 TWI Ltd., Granta Park, Great Abington, Cambridgeshire CB21 6AL, UK

**Keywords:** ultrasonic guided waves, above-ground storage tanks, tank floor inspection, numerical simulations, non-destructive testing

## Abstract

There is an increasing interest in using ultrasonic guided waves to assess the structural degradation of above-ground storage tank floors. This is a non-invasive and economically viable means of assessing structural degradation. Above-ground storage tank floors are ageing assets which need to be inspected periodically to avoid structural failure. At present, normal-stress type transducers are bonded to the tank annular chime to generate a force field in the thickness direction of the floor and excite fundamental symmetric and asymmetric Lamb modes. However, the majority of above-ground storage tanks in use have no annular chime due to a simplified design and/or have a degraded chime due to corrosion. This means that transducers cannot be mounted on the chime to assess structural health according to the present technology, and the market share of structural health monitoring of above-ground storage tank floors using ultrasonic guided wave is thus limited. Therefore, the present study investigates the potential of using the tank wall to bond the transducer instead of the tank annular chime. Both normal and shear type transducers were investigated numerically, and results were validated using a 4.1 m diameter above-ground storage tank. The study results show shear mode type transducers bonded to the tank wall can be used to assess the structural health of the above-ground tank floors using an ultrasonic guided wave. It is also shown that for the cases studied there is a 7.4 dB signal-to-noise ratio improvement at 45 kHz for the guided wave excitation on the tank wall using shear mode transducers.

## 1. Introduction

Structural Health Monitoring (SHM) of engineering structures using ultrasonic guided waves is a rising area of interest. Much research has been conducted on the use of ultrasonic guided waves to inspect elongated engineering structures, i.e., pipes, plates, rails, and cables, because of their inherent long range propagation [1]. Worlton [2] and Viktorov [3] explored the potential of Lamb and Rayleigh waves to inspect plate-like structures non-destructively and the market interest led to ultrasonic-guided wave testing being commercialized for pipe inspection in 1994 [4,5]. Commercial-guided wave testing systems have evolved vastly over the past two decades to fulfill many industry requirements. Ultrasonic-guided wave testing is adopted commercially to conduct SHM of elongated structures in industries, i.e., oil & gas, nuclear, renewable energy, and aerospace. The application of guided waves to assess the structural health of above-ground storage tank floors is an emerging technology [6]. Inspections of above-ground storage tanks are important to avoid leakage to the surroundings, causing catastrophic events and harm to the environment. In 2006, Chang et al. [7] conducted a study of storage tank accidents. The study showed 74% of accidents occurred in petro-chemical refineries, and 85% of the accidents have caused fire and explosions. Approximately 60% of the accidents are due to crack, rupture, and leakage.

Much research has been conducted on the inspection of above-ground storage tank floors using guided waves. The main difficulty with SHM using guided waves is the level of attenuation due to long-distance propagation requirements, as well as reflection and mode conversion of waves at boundaries. The fundamental symmetric Lamb mode has been used as the principal mode of interest due to low energy losses compared to the fundamental asymmetric Lamb mode [8]. The fundamental shear horizontal mode is an interesting alternative to the fundamental symmetric mode due to its non-dispersive characteristic. Another factor to consider is the welding seam, which reflects the transmitted signal and disturbs the propagation. To minimize this effect, a pitch-catch configuration is equipped in the data acquisition stage. Transducer attachment is also a problem as the tank perimeter gets heavily corroded over time due to environmental influences.

Heavy corrosion of the tank annular chime limits the market share for SHM of tanks using guided waves as the transducer bonding process becomes challenging. Therefore, this study investigates the potential for using the tank wall to bond transducers for SHM of tank floors using guided waves. Both normal and shear loads were considered in this study. A previously validated finite element method has been used to investigate the guided wave propagation across a tank floor [9]. The modes of interest for this application are thus the fundamental symmetric mode (normal load) and the fundamental shear horizontal mode (shear load). The paper is organized as follows: related work is given in Section 2, numerical simulations and results are presented in Section 3, and experimental validation is reported in Section 4, followed by conclusions in Section 5.

## 2. Related Work

### 2.1. Above-Ground Storage Tank Floor Inspection

Over the years, numerous Non-Destructive Testing (NDT) techniques have been used to inspect the condition of storage tanks, e.g., penetrant testing, magnetic particle testing, radiography, eddy current, thermography, acoustic emission, and conventional ultrasonic testing [10]. Inspection of storage tanks using these localized inspection techniques is time consuming and expensive, mainly due to the need to empty and clean the tank prior to inspection. The SHM of tank floor is more important compared to the tank wall, due to the fact that degradation of the tank floor is not visible until it becomes severe. In 2006, Mažeika et al. [6] studied the potential for using guided waves for storage tank floor inspection, which does not require emptying or cleaning the tank.

**Figure 1:**
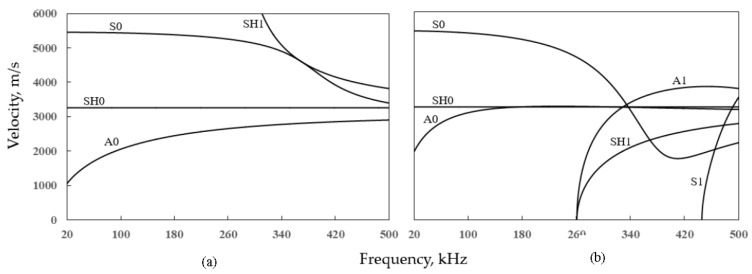
Dispersion curves for a 6.25 mm thick steel plate (**a**) phase velocity and (**b**) energy velocity.

Due to its layout, guided wave inspection of tank floor is complicated. A tank floor comprises a large number of plates (dependent on the tank diameter) of 6–8 mm thickness joined with lap welds. There are 3 fundamental wave modes in the guided wave operating frequency range (20–100 kHz) for plate inspection (see the dispersion curves in Figure 1): namely, the fundamental Symmetric Lamb mode, S0, the Asymmetric Lamb mode, A0, and the Shear Horizontal (SH) mode, SH0. Based on the availability of flexible commercial transducer (Macro Fibre Composite [11]), Mažeika et al. [12] investigated the use of the S0 mode for guided wave inspection of storage tank floor. This choice was informed by consideration of the low attenuation (due to the interaction with fluid inside the tank) of S0 compared to A0. The SH0 mode is a particularly interesting mode for this application due to its non-dispersive character (refer Figure 1) [13].

**Figure 2:**
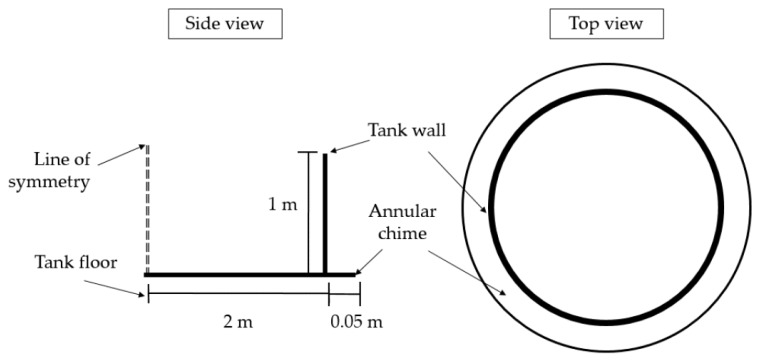
Schematics of the modelled tank, illustrating the nomenclature used in this study.

Environmental conditions, i.e., attenuation due to having fluid contact and elevated temperature, are significant challenges for tank floor inspection using guided waves. The impact of attenuation due to the contact with fluid has been studied by Yu et al. in 2015 [14] using a plate one-side immersed in fluid. The elevated temperature is also an important factor for guided wave propagation, which has been studied by many researchers. Strategies to compensate the temperature influence for guided wave inspection were reported by Croxford et al. in 2009 [15]. Considering the large coverage area and complexity of tank floor designs, guided waves should be transmitted with as much energy as possible. To achieve full coverage, a Pitch-catch configuration (through transmission) was used for data collection and the relevant transducer array layout was studied by Mažeika [6] and Feng [16]. Currently, normal mode transducers (elongated type) are installed on the annular chime (refer Figure 2) of the tank to transmit guided waves across the floor plate, and a tomographic technique is used to map the structural health of the tank floor [12]. However, shear mode transducers have not yet been investigated for guided wave inspection of storage tank floors due to the lack of flexible shear mode transducer availability.

### 2.2. Dispersion Curve Calculation for Above-Ground Storage Tank

In the ultrasonic guided wave operating frequency range, a number of fundamental modes can propagate in the tank floor and tank wall, and it is important to analyze mode shapes and wave velocities for these modes. For plate-like structures, Lamb waves have been extensively studied previously, and analytical solutions exist for Eigen analysis of these modes. The commercial software package DISPERSE is able to calculate dispersion curves for Lamb and shear-horizontal waves [17]. The global matrix technique is used to calculate the Eigen solutions. However, this technique is limited to simple waveguides, and mode searching is time consuming because an iterative procedure is used. Alternatively, an efficient Semi-Analytical Finite Element (SAFE) technique can calculate dispersion curves for waveguides of arbitrary cross-section [18]. This method introduces analytical modal expressions into finite element solutions, thus only meshing the cross-sectional area of the waveguide. For axisymmetric and plate-like structures, a one-dimensional Eigen problem can be further developed by making use of the symmetry or uniformity of the structure [19]. This will only mesh the waveguide in the radial or thickness direction. For large radius of curvature pipes (large outer radius compared to wall-thickness), dispersion curves for circumferentially propagating waves are identical to dispersion curves for Lamb waves in a flat plate of the same thickness and material properties [20]. Other numerical techniques are also available for dispersion analysis of waveguides with an arbitrary cross-section, such as the Scaled Boundary Finite Element Method, Wave Finite Element Method, Boundary Element Method, etc. [21,22,23]. In applications where the wave is propagating in one axis, SAFE can be further combined with the conventional finite element method to study wave scattering from a defect in a uniform waveguide [24].

The SAFE method has been used to calculate dispersion curves for Lamb and SH waves in the current tank floor studied here [18]. The thickness of the tank floor is 6.25 mm, and material properties are shear velocity $c_{T}=3260\;m/s$, Lamb velocity $c_{L}=5960\;m/s$, and density $\rho =8030\;\mathrm{kg}/m^{3}$. Three-noded line isoparametric elements are used to mesh the tank floor, and element size is 1 mm. The total number of degrees of freedom is 30. This ensures at least 13 nodes per wavelength for the shortest wave in the frequency range studied here. The SAFE model is programmed in MATLAB and executed on a laptop with a 2.6 GHz Intel^®^ Core™ CPU and 16 GB of RAM. The computation time is around 0.01 s per frequency, including the calculation of energy velocity.

Phase and energy velocities are presented in Figure 1. For the lossless system studied here, energy velocity equals group velocity [24]. Note that the SAFE method also delivers evanescent modes and backward waves. However, these modes are of no interest here and are not included. In the low frequency range, three fundamental Lamb and SH modes exist. It can be seen that up to 100 kHz, A0 is very dispersive and is thus not suitable for NDT applications. As frequency increases, S0 also becomes more and more dispersive. A1 and SH1 modes cut off near 260 kHz, and so it is preferable to limit the frequency range below 260 kHz for practical NDT applications. Note that the thickness and material properties of the tank wall are the same as the tank floor. However, the tank wall is slightly curved, and the curvature (ratio between radius and thickness of the wall) of the tank wall is 320. It is well known that dispersion curves for axisymmetric modes in a large curvature pipe approach dispersion curves for a flat plate [20]. To verify this, the influence of the tank wall curvature is examined. The SAFE method [24] is used to calculate dispersion curves for guided waves propagating in the axial direction of the tank wall, and these dispersion curves overlay dispersion curves for the flat-plate shown in Figure 1. For clarity in the figure, the overlaid dispersion curves are not shown. The dispersion curves shown in Figure 1 are thus used for both the tank floor and tank wall.

## 3. Numerical Investigation

### 3.1. Numerical Simulation

Finite Element Analysis (FEA) was performed to study the potential for guided wave excitation from the tank wall to assess the structural health of the tank floor. The FEA was performed in two separate parts; guided wave excitation from the tank floor to study the conventional operating conditions, and guided wave excitation from the tank wall as the proposed technique. A 3D model was built for the numerical analysis using ABAQUS/EXPLICIT version 6.13 [25,26]. A solid transient analysis was conducted, governed by Navier’s equation for an isotropic medium as follows [14],

(1) $$\left(\lambda +\mu \right)\nabla \left(\nabla \cdot u^{\prime }\right)+\mu \nabla^{2}u^{\prime }=\rho \frac{\partial^{2}u^{\prime }}{\partial t^{2}}$$

where λ and μ are Lamé constants, $u^{\prime }$ is the displacement vector, $\nabla^{2}$ is the Laplace operator, and ρ is the material density. The ABAQUS/EXPLICIT mode uses the central difference rule to integrate Equation (1) explicitly over time. At the commencement of the time increment, the program solves for dynamic equilibrium which states the nodal mass matrix, *M*, times the nodal acceleration, *ü*, as a property of nodal force as follows [25],

(2) Mü=P−I

where *P* is the external applied force, and *I* is the internal element force. The element acceleration at the commencement of the current time increment can be calculated as follows,

(3) $$ü{|}_{\left(t\right)}={\left(M\right)}^{-1}\cdot \left(P-I\right){|}_{\left(t\right)}$$

The acceleration is integrated over time using the central difference rule to calculate a change in velocity, which is then added to the velocity from the middle of the previous time increment to determine the velocity at the middle of the current time increment as follows,

(4) $$ü{|}_{\left(t+\frac{\Delta t}{2}\right)}=ü{|}_{\left(t-\frac{\Delta t}{2}\right)}+\frac{\left(\Delta t{|}_{\left(t+\Delta t\right)}+\Delta t{|}_{\left(t\right)}\right)}{2}\;ü{|}_{\left(t\right)}$$

Similarly, the velocity is integrated over time and added to the displacement $u^{\prime }$ at the commencement of the time increment to determine the displacement at the end of the increment as follows,

(5) $$u^{\prime }{|}_{\left(t+\Delta t\right)}=u^{\prime }{|}_{\left(t\right)}+\Delta t{|}_{\left(t+\Delta t\right)}ü{|}_{\left(t+\frac{\Delta t}{2}\right)}$$

A 4.1 m diameter steel tank was modelled with a 50 mm annular chime and tank wall height of 1 m. The thickness of the tank wall and floor was 6.25 mm. Material properties are given in the previous section (refer Section 2.2). Figure 2 illustrates the schematics of the modelled tank.

**Figure 3:**
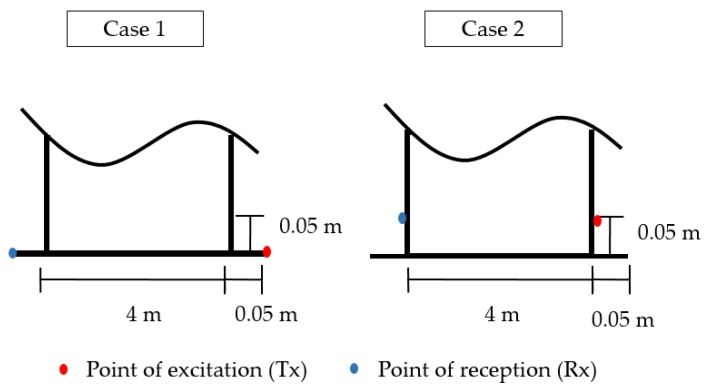
Layout of the Finite Element Analysis (FEA) model and the point of excitation and reception of both the cases studied. **Case 1**: excitation and reception from the tank floor; and **Case 2**: excitation and reception from the tank wall.

The layout of the Finite Element (FE) model and the main cases studied in the FEA are illustrated in Figure 3, showing excitation and reception from the floor (Case 1) and wall (Case 2). Excitation points were selected 50 mm from the annular chime edge between tank wall and floor, and reception points were in opposite positions. The mesh element sizes (*h*_0_) were in the range of 3.125–3.13 mm and calculated as follows,

(6) $$h_{0}=\frac{c}{Nf_{0}}$$

where c is the velocity of the slowest mode, N is the number of elements per wavelength, and $f_{0}$ is the frequency of interest.

The ABAQUS element type C3D8R (linear eight node brick elements with reduced integration) was used to achieve efficient computation time, and the mesh refinement was such that there were at least eight elements to represent the smallest possible wavelength in the main lobe of the frequency bandwidth. This level of mesh refinement was validated in previous studies [9,27]. The excitation tone-burst was a 60 kHz 10-cycle Hann-windowed pulse. Both normal and shear mode loads were investigated in this study to generate fundamental Lamb modes and shear modes at the direction of interest (towards the point of reception). Theoretical wave propagation directionality of a point source vibrating in normal and shear directions is documented in the literature [28], which illustrates that Lamb modes propagate in the plane of vibration and shear modes propagate perpendicular to the plane of vibration.

### 3.2. Numerical Results

**Figure 4:**
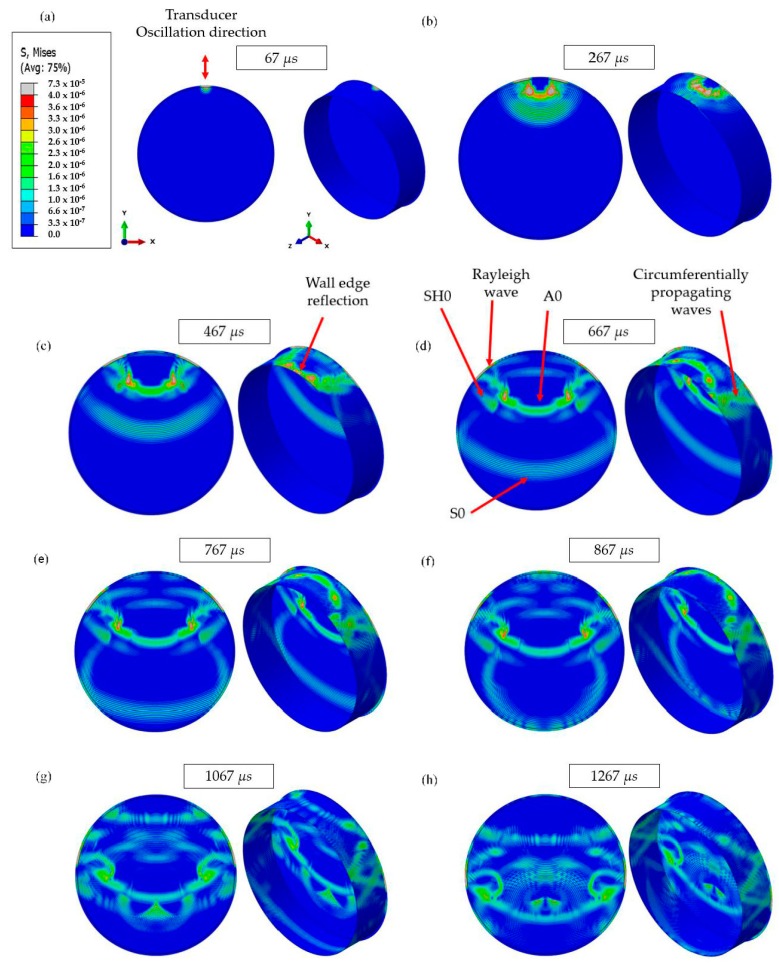
FEA results: wave propagation at different time increments on a steel 4.1 m diameter, 1 m wall tank at 60 kHz (the tank annular chime is used as the point-of-excitation as illustrated in Figure 3—case 1), and the color scale represents the Von-Mises stress. (**a**) 67 µs, (**b**) 267 µs, (**c**) 467 µs, (**d**) 667 µs, (**e**) 767 µs, (**f**) 867 µs, (**g**) 1067 µs, (**h**) 1267 µs.

**Figure 5:**
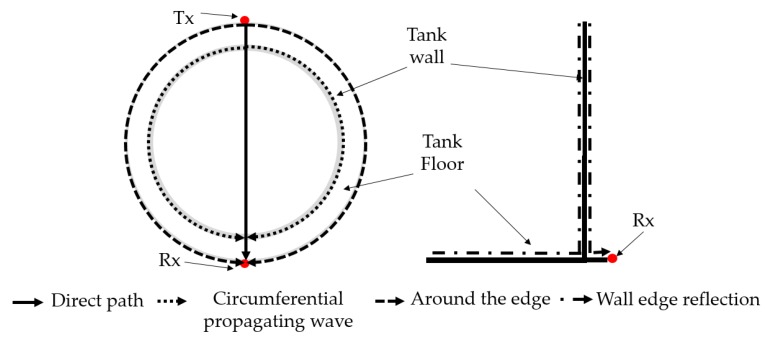
Primary wave paths for wave propagation in the tank.

This is a complex structure to be analyzed, and numerical results for waves propagating at different time increments for the normal mode excitation on the tank floor are illustrated in Figure 4. It can be seen that multiple wave paths exist, which makes the analysis complicated. Figure 5 illustrates the primary wave paths of a tank which can be used to interpret the time-domain data. Time-of-Arrival (ToA) data for the wave paths in the current study (4.1 m diameter tank) for each of the fundamental modes are summarized in Table 1. The primary direct wave path is across the tank floor. The transmitter is located on the tank wall, and there is a right angle bending between the tank wall and tank floor where the welding seam is. This does not change wave velocity, however; partial wave energy of the incident wave could be scattered by the bending. The secondary wave path is from the transducer to the top of the tank wall, where the wave is reflected back to the tank floor and then travels across the tank floor. The Rayleigh wave travels around the annular chime edge of the tank floor. Waves also travel circumferentially around the tank wall, however; these waves are scattered by the edges of the tank wall, and travel to the receiver at a later time, and can be truncated (the wave paths are illustrated in Figure 5). All fundamental modes are generated in both normal and shear applied load but with different amplitudes based on mode sensitivity for the mode of vibration [29,30].

**Figure 6:**
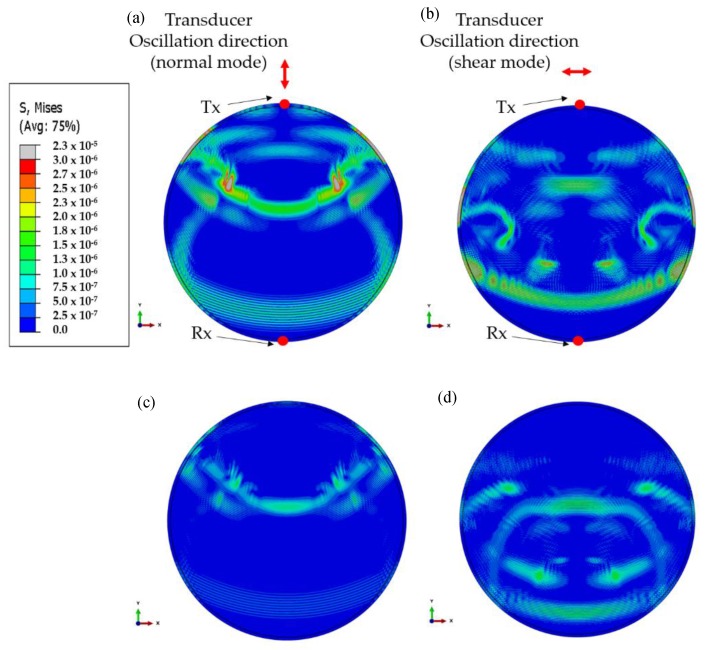
FEA results (Von Mises stress) of ultrasonic guided wave excitation on tank annular chime and tank wall: applied (**a**) normal stress; (**b**) shear stress on tank chime; (**c**) normal stress; and (**d**) shear stress on tank wall.

**Figure 7:**
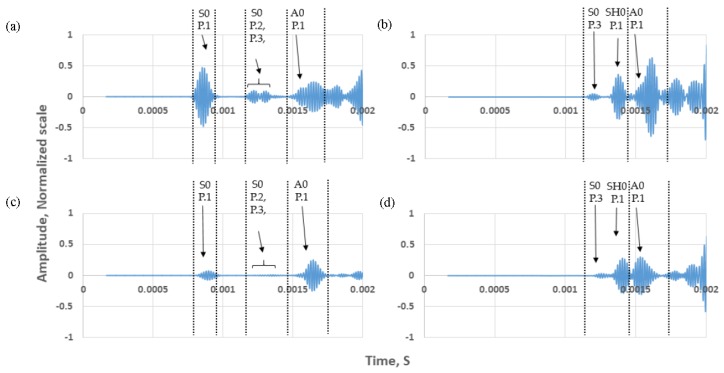
Normalized time-domain results for both excitation on the tank annular chime and wall; monitored waveforms are labelled according to the Path ID in Table 1. Case 1: (**a**) normal mode, (**b**) shear mode; and Case 2: (**c**) normal mode, (**d**) shear mode. Results are normalized to the maximum of all 4 signals.

The Von-Mises FEA results for both studies (wave excitation on tank annular chime and tank wall) are illustrated in Figure 6 for the mode of interest (S0 and SH0 for normal mode and shear mode excitations, respectively). The wave propagation behavior for each case is illustrated and a good correlation with wave paths can be seen in Figure 5. Monitored time-domain signals at the reception point are documented in Figure 7. Received signals are labelled and identified using the ToA tabulated in Table 1.

Table 2, summarizes the maximum amplitude of the direct path (Path ID P.1) signals for the mode of interest for each case studied. There is an amplitude drop of 18 dB at 60 kHz for the applied normal load on the tank wall compared to the tank floor, but only a 2 dB amplitude drop for the shear load. This shows that the application of shear stress on tank wall has potential to be used in guided wave testing of tank floors.

## 4. Experimental Validation

**Figure 8:**
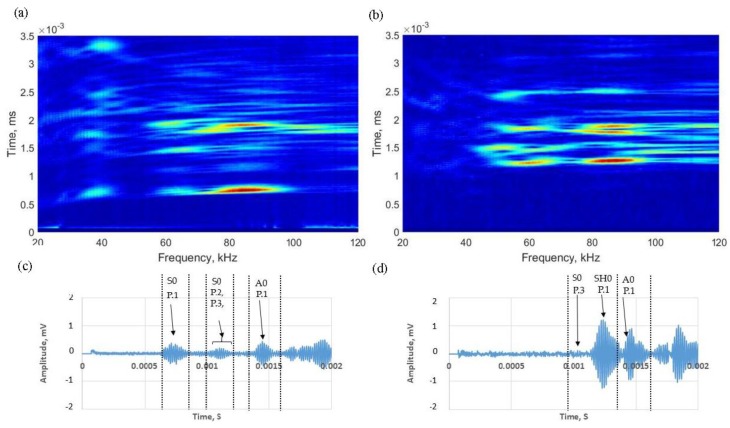
Experimental results: contour plot of the frequency sweep (**a**) in-plane; (**b**) shear transducers attached on the tank floor to excite guided waves for tank floor inspection with corresponding time-domain representation at 60 kHz; (**c**) in-plane; and (**d**) shear.

**Figure 9:**
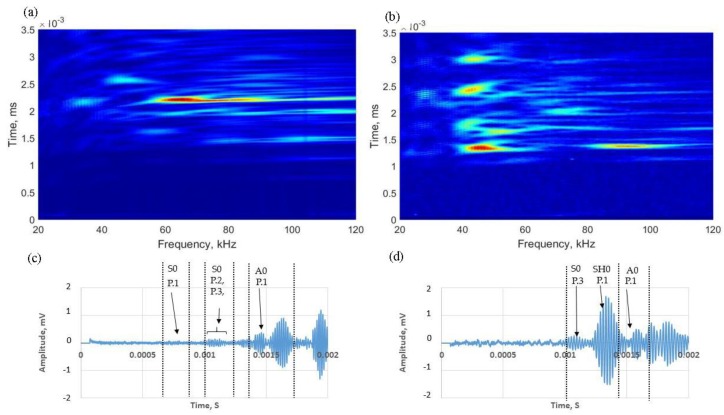
Experimental results: contour plot of the frequency sweep (**a**) in-plane; (**b**) shear transducers attached on the tank wall to excite guided waves for tank floor inspection with corresponding time-domain representation at 45 kHz; (**c**) in-plane; (**d**) shear.

Experiments were conducted on a steel tank to validate the FEA results in Section 3.2. The tank has the same dimensions as modelled for the numerical analysis (4.1 m diameter, 6.25 mm plate thickness), and the experimental setup is as illustrated in Figure 3. Both normal and shear transducers were used in this study, driven by the guided wave testing system, Teletest Focus+ [5]. Transducers were bonded to the surface using an Acrylic adhesive, and a magnet with 90 kg pulling force has been used to apply load over the curing process. Data collection was in a pitch-catch configuration in order to identify excited modes discretely, as in the simulation. A frequency sweep was conducted 20–120 kHz in 1 kHz increments. Hann-windowed pulse modulation was used to excite a discrete input signal. Figure 8 shows a contour plot of the acquired amplitude data (frequency range of 20–120 kHz), and a time domain representation at 60 kHz for Case 1 (guided wave excitation on the tank floor) and Case 2 (guided wave excitation on the tank wall) is shown in Figure 9. There is good correlation between the experimental and numerical results, giving confidence in the numerical simulation analysis in Section 3.2. However, some of the waveforms are buried in the noise flow due to low amplitude compared to the numerical simulation (e.g., Path ID P.3 S0 mode in Figure 8b). The received waveforms were labelled and identified based on their ToA listed in Table 1.

The Signal-to-Noise Ratio (SNR) can be used to compare the transducer performance for the studied cases, calculated as,

(7) $$SNR=20\log_{10}\left(\frac{S_{Max}}{N_{Max}}\right)$$

where $S_{Max}$ is the maximum amplitude of the mode of interest, and $N_{Max}$ is the maximum of the noise range.

SNR for a range of frequencies is summarized in Table 3. Based on these results, the normal mode transducers are unsuitable for guided wave excitation on the tank wall. The SNR drop variation is in the range 7.7–17.4 dB. Summarizing the investigated cases, the shear mode type transducers are suitable for guided wave excitation on the tank wall to inspect the tank floor. At 45 kHz, there is a 7.4 dB improvement in SNR compared to guided wave excitation on the tank floor. Theoretical, numerical, and experimental ToA comparison is tabulated in Table 4. Based on the results, there is less than 5% error in this study.

## 5. Conclusions

SHM of above-ground tank floor inspection using guided waves is a growing area of interest. It is a non-invasive and economically viable means of assessing structural degradation. Currently, normal-stress type (elongated type) transducers are attached to the tank annular chime to excite guided waves to assess the structural health of the tank floor. The present study investigates the potential of using the tank wall to excite guided waves as an alternative to the annular chime, which is desirable because, over time, corrosion of the annular chime can impede transducer installation; it is also desirable for accommodating new tank designs. Both normal and shear type transducers were considered in the current study. It was demonstrated that the normal mode transducers cannot be applied to excite guided waves from the tank wall to inspect the floor, and in this case the elongated type of transducer is not applicable. Shear type transducers have shown merit in exciting guided waves from the wall. At 45 kHz, there is a 7.4 dB SNR improvement compared to the guided wave excitation from the floor. Furthermore, based on the ToA comparison of theoretical, numerical, and experimental results of the mode of interest, there is only 5% error. Further work needs to be conducted to construct a tomographic image of the tank floor using the transducers attached to the wall and also to study the defect sensitivity.

## Figures and Tables

**Table 1:** Wave paths and analytical Time-of-Arrival (ToA) of potential signals for wave excitation on the tank floor.

Path ID	Path	Distance (m)	ToA ^1^
P.1	Direct path	4.1	S0—759 µs
			SH0—1281 µs
			A0—1576 µs
P.2	Wall edge reflection	6.1	S0—1138 µs
			SH0—1921 µs
			A0—2365 µs
P.3	Circumferential propagating waves	6.3	S0—1166 µs
P.4	Around the edge (Rayleigh wave)	6.5	2241 µs

^1^ Based on the dispersion curves in Figure 1, velocity of S0—5.4 km/s, SH0—3.2 km/s, A0—2.6 km/s.

**Table 2:** Amplitude of each mode of interest for the both cases studied in the numerical analysis.

Case	Mode of Interest	Mode of Excitation	Normalized Amplitude
1	S0	Tank floor	0.43
2	S0	Tank wall	0.05
1	SH0	Tank floor	0.33
2	SH0	Tank wall	0.25

**Table 3:** Experimental SNR of the mode of interest for both cases for a range of frequencies.

Frequency (kHz)	SNR (dB)		Variation (dB)	SNR (dB)		Variation (dB)
	Case 1-S0	Case 2-S0		Case 1-SH0	Case 2-SH0	
40	16.5	5	−11.5	8.4	13.8	5.4
45	9.2	1.4	−7.8	13.7	21.1	7.4
50	8.1	0.4	−7.7	15.9	17.4	1.5
55	8.9	0.9	−8	19.1	16.9	−2.2
60	13.1	1.9	−11.2	18.1	12.4	−5.7
65	18.9	1.5	−17.4	17.6	9.3	−8.3
70	17.5	4.2	−13.3	11.1	6.4	−4.7

**Table 4:** Comparison of theoretical, numerical, and experimental ToA of modes of interest (P.1—direct path).

Frequency (kHz)	Theoretical (µs)	Numerical (µs)	Experimental (µs)	Theoretical to Numerical Error (%)	Theoretical to Experimental Error (%)
S0	759	776	723	2.2	4.7
SH0	1281	1299	1242	1.4	3
A0	1576	1593	1533	1	2.7
